# Spatial distribution of arboviruses and its association with a social development index and the waste disposal in São Luís, state of Maranhão, Brazil, 2015 to 2019

**DOI:** 10.1590/1980-549720240017

**Published:** 2024-05-03

**Authors:** Emile Danielly Amorim Pereira, Cleber Nascimento do Carmo, Waleska Regina Machado Araujo, Maria dos Remédios Freitas Carvalho Branco

**Affiliations:** I Fundação Oswaldo Cruz, Sergio Arouca National School of Public Health – Rio de Janeiro/RJ, Brazil.; II Secretaria de Estado da Saúde do Maranhão – São Luís (MA), Brazil.; III Universidade Federal do Maranhão, Graduate Program in Public Health – São Luís (MA), Brazil.

**Keywords:** Chikungunya, Dengue, Zika, Solid waste, Ecological studies, Chikungunya, Dengue, Zika, Resíduos sólidos, Estudos ecológicos

## Abstract

**Objective:**

To detect spatial and spatiotemporal clusters of urban arboviruses and to investigate whether the social development index (SDI) and irregular waste disposal are related to the coefficient of urban arboviruses detection in São Luís, state of Maranhão, Brazil.

**Methods:**

The confirmed cases of Dengue, Zika and Chikungunya in São Luís, from 2015 to 2019, were georeferenced to the census tract of residence. The Bayesian Conditional Autoregressive regression model was used to identify the association between SDI and irregular waste disposal sites and the coefficient of urban arboviruses detection.

**Results:**

The spatial pattern of arboviruses pointed to the predominance of a low-incidence cluster, except 2016. For the years 2015, 2016, 2017, and 2019, an increase of one unit of waste disposal site increased the coefficient of arboviruses detection in 1.25, 1.09, 1.23, and 1.13 cases of arboviruses per 100 thousand inhabitants, respectively. The SDI was not associated with the coefficient of arboviruses detection.

**Conclusion:**

In São Luís, spatiotemporal risk clusters for the occurrence of arboviruses and a positive association between the coefficient of arbovirus detection and sites of irregular waste disposal were identified.

## RESUMO

**Objetivo:**

Detectar aglomerados espaciais e espaço-temporais de arboviroses urbanas e investigar se o índice desenvolvimento social (IDS) e o descarte irregular de lixo estão relacionados ao coeficiente de detecção das arboviroses urbanas em São Luís, Maranhão.

**Métodos:**

Os casos confirmados de dengue, Zika e chikungunya em São Luís, no período de 2015 a 2019, foram georreferenciados para o setor censitário de residência. O modelo de regressão Autorregressivo Condicional Bayesiano foi utilizado para identificar a associação entre o coeficiente de detecção de arboviroses urbanas, IDS e pontos de descarte irregular de lixo.

**Resultados:**

O padrão espacial de arboviroses apontou para a predominância de cluster de baixo coeficiente de detecção, exceto em 2016. Para os anos de 2015, 2016, 2017 e 2019, o aumento de uma unidade de ponto de lixo aumenta o coeficiente de detecção de arboviroses em 1,25, 1,09, 1,23 e 1,13 casos de arboviroses por 100 mil habitantes, respectivamente. O IDS não foi associado ao coeficiente de detecção de arboviroses.

**Conclusão:**

Em São Luís foram identificados aglomerados espaço-temporais de risco para a ocorrência de arboviroses e a associação positiva entre o coeficiente de detecção de arboviroses e os pontos de descarte irregular de lixo.

## INTRODUCTION

Arboviruses are a growing public health issue in the world^1^. In Brazil, the simultaneous occurrence of the three urban arboviruses (Dengue, Chikungunya, and Zika) is an important challenge for the Brazilian Unified Health System, both because of the magnitude and severity of the cases and because of the difficulty of differential diagnosis. These diseases have a wide geographical distribution and are present in most municipalities and in the five macroregions of the country^2^.

Several studies^3-5^ indicate that the absence or insufficient provision of sanitation services contribute to the production of spaces suitable for the maintenance of *Aedes (Stegomyia) aegypti* (Linnaeus, 1762), the main vector of urban arboviruses. Urban spaces with inadequate and insufficient sanitation may concentrate individuals who are more vulnerable to the infection with viruses transmitted by vectors, such as *A. aegypti*, due to greater exposure to mosquitoes and reduced access to environmental and personal prevention measures^6^.

One of the main sanitary measures that help prevent the proliferation of diseases in urban and rural areas is waste collection^7-11^. This is because household and urban waste provide favorable conditions for the larval development of *A. aegypti* ^12^. For instance, studies conducted in Australia^13^ and Brazil^4^ pointed to the existence of a large volume of positive breeding sites derived from household waste.

In Brazil, local governments are responsible for managing solid waste produced in the cities. However, in 2008, 50.8% of Brazilian municipalities destined their waste for open-air disposal sites (dumps), according to the National Basic Sanitation Survey of the Brazilian Institute of Geography and Statistics (IBGE)^14^. The Northeast region registered the highest proportions of this waste destined for landfills (89.3%) and the state of Maranhão ranked third (96.3%)^14^.

In São Luís, capital of the state of Maranhão, the intense and disordered process of urban expansion, fueled by real estate speculation and irregular occupations, has intensified this issue^15^. Urban waste collection is precarious and dumps are present throughout the city^16^. The increase in solid waste and the inadequate disposal of waste has contributed to the proliferation of these irregular disposal sites.

The universalization of access to the basic sanitation service in São Luís remains a challenge, which can be overcome by improving solid waste management. Thus, identifying a pattern of the distribution of arboviruses within the municipality and a possible association with social development and with irregular waste disposal sites is necessary to propose measures to prevent diseases related to urbanization.

In this study we aim to detect spatial and spatiotemporal clusters of urban arboviruses (Dengue, Chikungunya, and Zika) and to identify whether social development and waste management are related to the coefficient of detection of these diseases in the census tracts of São Luís, Maranhão, in the period from 2015 to 2019.

## METHODS

**Figure 1:**
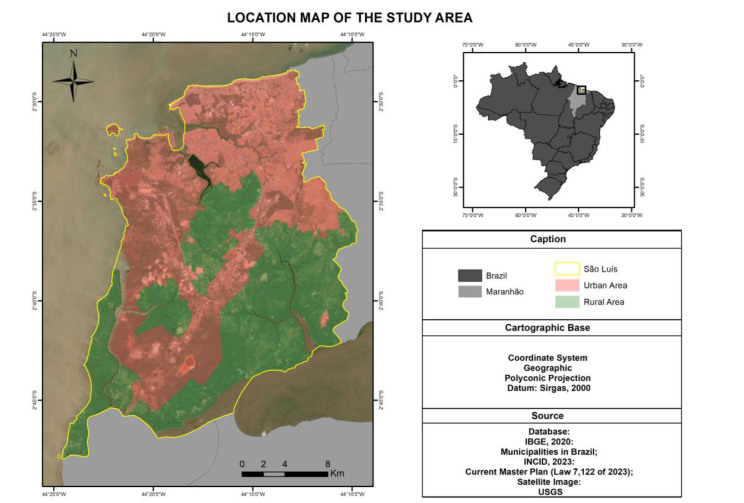
Location map of the municipality of São Luís, with representation of urban and rural areas.

Source: Master Plan for the municipality of São Luís for the year 2019. Available at: https://www.saoluis.ma.gov.br/incid/conteudo/4023. Cited on: Nov. 28, 2023.

An ecological study was conducted in São Luís, whose estimated urban population was 1,115,932 inhabitants and the population density was 1,215.69 inhabitants/km² in 2021^17^. The units of analysis were the census tracts of the municipality, totaling 1,126 censuses^2^ (Figure 1).

The study population comprised all cases reported in the Notifiable Diseases Information System (*Sistema de Informação de Agravos de Notificação* – SINAN) of Dengue, Chikungunya, and Zika, confirmed by clinical epidemiological or laboratory criteria, of residents of São Luís, from January 1st, 2015 to December 31st, 2019. The case definitions followed the protocols of the Brazilian Ministry of Health^18^.

To identify arboviruses cases, the variables “municipality of residence,” “date of notification,” and “final classification (discarded, Dengue, Chikungunya, and Zika)” were considered. Discarded cases under investigation, imported from other municipalities and without information on the variables “municipality of residence” and “final classification,” were excluded.

SINAN data were georeferenced to the census tract of residence, using the fields “address” and “number” that were compared on the platforms: *Google Maps, Bing Maps, and Wikimapia* in order to identify the geographical coordinates (x, y) of latitude and longitude, using the nearest address. Geographic coordinates were entered in the Geocoding tool in the ArcGIS 10.4.1 software. Subsequently, the occurrences were converted into a cartographic base of points using the Geographic Information System (GIS), Qgis version 3.10.

Data on the 600 irregular waste disposal sites, in the years 2015, 2016, 2017, and 2019, came from the Municipal Department of Public Works and Services (*Secretaria Municipal de Obras e Serviços Públicos* – SEMOSP), mapped through the municipality’s Public Cleaning Superintendence. Data for the year 2018 were not available, so the number of irregular waste disposal sites for that year was obtained by calculating the average for the years 2017 and 2019.

The Social Development Index (SDI) of São Luís was calculated, by census tract, based on the methodology proposed by Cavallieri and Lopes^19^. It is an aggregated social index based on indicators from the 2010 census (access to basic sanitation, housing quality, education, and income), with the purpose of measuring the degree of social development between geographical areas of the same nature, ranging from 0 to 1, with 0 being the worst condition of social development and 1 the best. In this study, the same four dimensions of analysis and variables as in the aforementioned study^19^ were used, except the education and income dimensions, as adjustments had to be made to these variables due to their unavailability in the 2010 IBGE Demographic Census.

- In the education dimension, only the variable percentage of illiteracy among people over 15 years of age was used;
- In the income availability dimension, the variable percentage of heads of household with an income of up to two minimum wages was replaced by the percentage of heads of household with an income of 5 to 10 minimum wages, considering that, among the categories, this was the lowest available income range.

The coefficients of urban arboviruses detection were estimated based on the division between the total number of cases of the three diseases reported in the census tract and the population of the respective census tract, multiplied by 100 thousand inhabitants (inhab.). The date of notification of the cases was considered for all the studied years. Population estimates by census tract were obtained from IBGE^20^.

For the spatial distribution of the coefficient of urban arboviruses detection, stratification was adopted based on the risk of occurrence of arboviruses used by the National Dengue Control Program (*Programa Nacional de Controle da Dengue*): low risk (up to 100 cases/100 thousand inhab.); moderate risk (101 to 300 cases/100 thousand inhab.); high risk (301 to one thousand cases/100 100 thousand inhab.); and epidemic (above one thousand cases/100 thousand inhab.)^21^.

The neighborhood matrix, first-order queen-type, was built and spatial dependence was measured. Pseudosignificance tests were calculated for 999 permutations. Spatial dependence can be expressed by spatial autocorrelation, which indicates how much a variable varies depending on its neighbors.

For spatial autocorrelation, the Global (Moran’s I) and Local Moran’s Indices were used. The Global Moran’s I can vary between -1 (negative spatial autocorrelation) and 1 (present a spatial pattern), and for data with no spatial dependence or very low spatial dependence, the value is close to zero^22^.

In order to highlight the places where spatial dependence was most pronounced, according to the census tracts, and to thoroughly verify the different association regimes formed, the Local Indicators of Spatial Association (LISA) was used, which indicates a value referring to the correlation of municipalities with their neighbors, pointing out where spatial autocorrelation has statistical significance. The analysis of the quadrants of the diagram indicates areas of positive spatial association Q1 (positive values, positive averages) and Q2 (negative values, negative averages) and areas of negative spatial association Q3 (positive values, negative averages) and Q4 (negative values, positive averages). In both indices (Global and Local), a value of p<0.05 was considered statistically significant.

To identify spatiotemporal clusters, the Kulldorff statistical scanning technique was used using the SatScanTM software. The Kulldorff scanning method simultaneously detects clusters in space and time, testing statistical significance, estimating the relative risk of each cluster^23^. As the variable of interest is the number of cases in a location, the expected number of cases *E*[*c*] in each cylinder follows a Poisson distribution given by (Equation 1):

Equation 1:

$$E\left[c\right]=CP\times p,$$

Where:

- C is the total number of cases;
- P is the total population;
- p is the population within the area of the cylinder.

Statistical significance is obtained from Monte Carlo simulations. The null hypothesis is rejected when less than 5% of the simulated values are greater than the observed value.

The relative risk (RR) obtained using the SatScanTM software is based on the difference between the temporal progression of the epidemic in the area compared to the overall progression estimated by the offset. Thus, the relative risk of each **cluster** is given by (Equation 2):

Equation 2:

$$RR=\frac{c/E\left[c\right]}{\left(C-c\right)/\left(C-E\left[c\right]\right),}$$

The Baysian Conditional Autoregressive model (CAR Bayes model) was used to verify the relationships between the coefficient of urban arboviruses detection and the SDI variables and irregular waste disposal sites, which incorporates the spatialization of the data in the estimation of the coefficient of detection adjusted to a single parameter.

The null hypothesis is that the standardized difference between the average of the first decile of the iterations and the average of the last fifth deciles follow a standard normal distribution. To diagnose the model, maps of the residues were drawn in the final CAR Bayes model, seeking evidence of a rupture of independence assumptions, that is, the presence of correlated errors. The Geweke method was used to analyze the convergence of the chains^24^.

To diagnose the model, maps of the residues were produced in the final model, seeking evidence of the rupture of independence assumptions, that is, a high concentration of positive or negative residues in a part of the map would indicate the presence of spatial autocorrelation.

The study was approved by the Research Ethics Committee of the National School of Public Health/Fundação Oswaldo Cruz, under opinion No. 4.510.977, CAEE No. 403558620.6.0000.5240.

The statistical modeling analyses and the production of the maps were carried out using the R Core Team statistical software, version 4.2.1.

## RESULTS

In São Luís, from 2015 to 2019, 40,353 cases of urban arboviruses (Dengue, Chikungunya, and Zika) were reported, with 846 cases excluded due to the lack of complete information on place of residence. Thus, we analyzed 39,507 cases of these diseases in this study.

**Figure 2:**
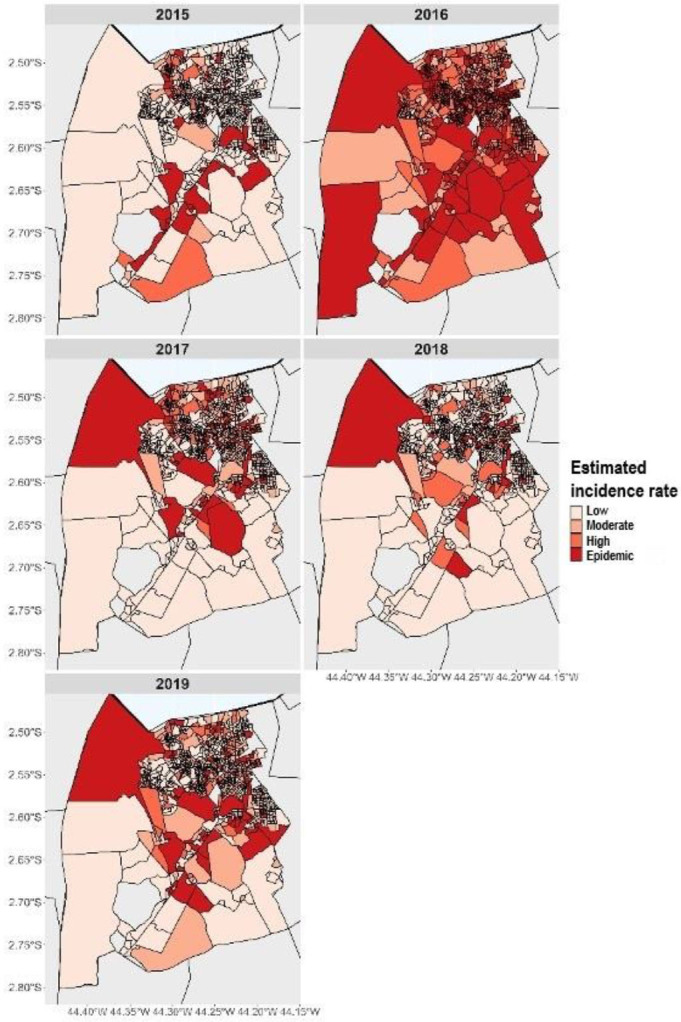
Map of the estimated coefficient of arboviruses detection in São Luís, Maranhão, Brazil (2015 to 2019).

Regarding the pattern of the distribution of the coefficient of urban arboviruses detection, we observed that census tracts varied from low coefficient of detection to epidemic census tracts. In the years 2015, 2017, 2018, and 2019, there was a predominance of areas with low coefficient of detection, with 880, 598, 776, and 582 census tracts, respectively. However, in 2016, we observed that there was an increase in census tracts with a moderate (n=255), high (n=398), and epidemic (n=435) coefficients of detection throughout the city of São Luís (Figure 2).

We observed that the highest coefficients of arbovirus detection predominated in census tracts located in the north of the city and in some rural municipalities. It is worth noting that a census tract, located in the rural area of the municipality, was epidemic in almost the entire studied period (Figure 2).

**Figure 3:**
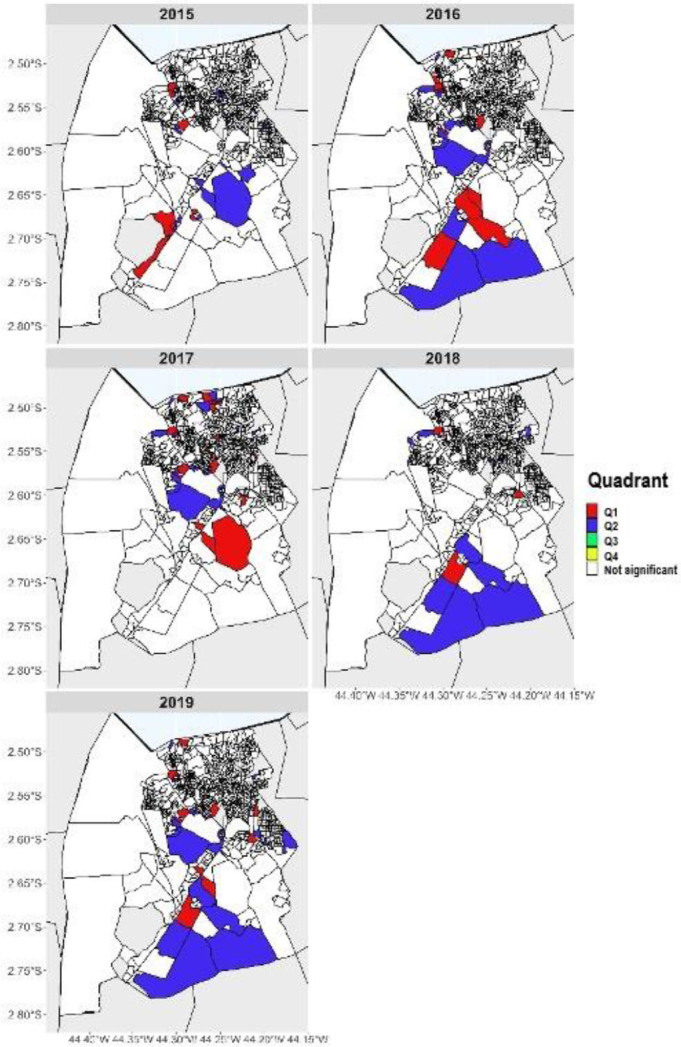
Local Moran’s Index for the coefficient of urban arboviruses detection in São Luís, Maranhão, Brazil (2015 to 2019).

With regard to the Local Moran’s Index, after comparing the Box Maps, the municipality was characterized by low-low clusters (the coefficient of urban arboviruses detection is low and its neighbors also have low coefficients), especially in rural census tracts, and high-high clusters (high coefficient of urban arboviruses detection, and their neighbors from census tracts also have high coefficients) in census tracts located in the central region and in the rural area (Figure 3).

With regard to the Local Moran’s Index, after comparing the Box Maps, the municipality was characterized by cluster areas with negative coefficient of arboviruses detection and average of their neighbors (Q2), especially in rural census tracts. Conversely, in neighborhoods located in the central area of the municipality and in the rural area, the opposite occurred: cluster areas that present positive values of the coefficient of detection and the average of their neighbors (Q1). We observed no areas considered as transition areas, located in quadrants Q3 (areas with a high proportion, surrounded by areas with a low proportion of this indicator) and Q4 (areas with a low proportion, surrounded by areas with a high proportion of the same indicator). This situation indicates that the areas belonging to these quadrants (Q3 and Q4) do not follow the same process of spatial dependence as the others (Figure 3).

In the spatiotemporal scanning statistics, it was possible to detect three statistically significant clusters. The population size of cluster 1 was 436,848 inhabitants, and 19,111 cases of arbovirus disease were detected in the 2015-2019 period. In cluster 2, the population size was 731 inhabitants and 1,216 cases of arboviruses were observed. In cluster 3, the population size was 28,077 inhabitants and 2,205 cases of arboviruses were observed. Clusters 2 and 3 were detected in the 2015-2017 period. Cluster 1 presented the lowest risk (RR=2.58) and the highest number of census tracts (n=462). Meanwhile, cluster 2 presented the highest relative risk (RR=71.76) and the lowest number of census tracts (n=2), all located in northern São Luís. The characteristics of these clusters were presented in Table 1.

**Table 1:** Characteristics of clusters that are statistically significant regarding the risk for arboviruses, according to the spatiotemporal scanning statistics, in the census tracts of the city of São Luís, Maranhão, Brazil, 2015–2019.

Diseases	Cluster	Period	Population	Number of census tracts	Number of observed cases	Number of expected cases	Relative risk	p-value
Arboviruses	1	2015–2019	436,848	462	19,111	10,437	2.58	<0.01
	2	2015–2017	731	2	1,216	17.47	71.76	<0.01
	3	2015–2017	28,077	32	2,205	670.85	3.42	<0.01

The census tracts with the highest RR were from the neighborhoods Vila Itamar (RR=113.88), Centro (RR=101.09), Maracanã (RR=87.92), Quebra Pote (RR=78.95), and Ribeira (RR=39.02), located in the north of the municipality.

According to the CAR Bayes regression model, irregular waste disposal sites are significant in the coefficient of arboviruses detection in the census tracts for all years, except 2018, as the credibility interval included zero. For the years 2015, 2016, 2017, and 2019, the increase of one unit of waste disposal site increased by 1.25, 1.09, 1.23, and 1.13 cases of arbovirus diseases per 100 thousand inhabitants, respectively. However, SDI was not significant in any of the surveyed years (Table 2).

**Table 2:** Bayesian Conditional Autoregressive regression model for the association of irregular waste disposal sites and the social development index with the coefficient of urban arboviruses detection in the census tracts of São Luís, Maranhão, Brazil, 2015–2019.

Year		Coefficient	Credibility interval 2.5%	Credibility interval 97.5%	Geweke
2015	Intercept	-21.1	-21.5084	-20.6532	-0.7
	Irregular waste disposal site	0.23	0.0405	0.3951	0.5
	Social Development Index	4.87	-2.2869	7.1241	0.6
	τ^2^	1.55	0.8962	2.1922	-0.7
	σ^2^	4.21	3.3833	4.9287	1.2
2014	Intercept	-16.3	-16.611	-16.0988	1.8
	Irregular waste disposal site	0.09	0.0124	0.1612	-0.3
	Social Development Index	-0.97	-2.5515	0.7252	-1.8
	τ^2^	2.09	1.6347	2.5467	0.1
	σ^2^	1.21	1.0611	1.3063	-0.9
2017	Intercept	-18.9	-19.4025	-18.5174	-1.5
	Irregular waste disposal site	0.21	0.0854	0.3358	0.3
	Social Development Index	0.37	-1.8229	3.5164	1.5
	τ^2^	2.85	2.2709	3.65	1.1
	σ^2^	2.43	2.1287	2.7742	1.3
2018	Intercept	-19.5	-20.2126	-18.8483	-1.8
	Irregular waste disposal site	0.15	-0.0067	0.2698	0.4
	Social Development Index	0.01	-3.2492	3.2097	1.7
	τ^2^	2.12	1.3949	2.928	-1.4
	σ^2^	2.97	2.4746	3.618	1.4
2019	Intercept	-18.7	-19.1155	-18.3119	-1.5
	Irregular waste disposal site	0.13	0.0051	0.2257	0.4
	Social Development Index	0.21	-2.0127	2.2888	1.3
	τ^2^	0.78	0.4842	1.1929	-0.9
	σ^2^	3.05	2.7189	3.4039	0.6

Note: τ^2^ = Kendall’s τ squared correlation coefficients. σ^2^= Variance.

In the analysis of the residues of the CAR Bayes model, we observed that the means and standard deviations of the errors were close to zero and all the models presented uncorrelated errors, meeting the assumption of error independence. The Moran’s index showed the absence of spatial autocorrelation for all the studied years: 2015 (I=-0.00; p=0.62), 2016 (I=-0.03; p=0.96), 2017 (I=-0.02; p=0.88), 2018 (I=0.02; p=0.07), and 2019 (I=0.06; p=0.06) (data not shown in the table).

## DISCUSSION

In our study, the spatial pattern of arboviruses found pointed to the predominance of clusters with a low coefficient of detection, except for the year 2016. Dengue is endemic in the city of São Luís. As of 2015, with the circulation of Chikungunya and Zika, the lack of immunity of the population, never previously exposed to the two viruses, and the high and prolonged viremia, increased the possibilities of transmission^25^.

One reason that may explain the high number of areas with low coefficients of detection for arboviruses is the underreporting of cases, a phenomenon that is still common in Brazil. Overall, notifications result from information provided by infected people seeking healthcare services. However, many cases may not have been reported for different reasons such as diagnostic errors, asymptomatic infections, problems accessing healthcare services^26^, among others. Several authors^27-30^ mentioned the underreporting of diseases as a limitation in their studies, which impairs the presentation of real circulation force and epidemiological magnitude, compromising the direction of disease control actions. In addition to this issue is the worsening of the underreporting of arboviruses cases due to the context of the new coronavirus (COVID-19) pandemic, observed by the significant reduction in the reporting of cases and deaths of these diseases as of 2020^2,31^.

In our results, we identified three spatiotemporal clusters as being at risk for the occurrence of arboviruses in São Luís from 2015 to 2019. The census tracts with the highest risks are located in the north of the municipality and in the rural area. Regarding the characteristics of these places, it should be noted that the north of the municipality is where the oldest neighborhoods and peripheral areas of São Luís are located, with the largest urban populations and population density, with an unplanned urbanization^32^. In the rural area of the municipality, a significant portion of the growth occurred in the form of subdivisions with uneven patterns.

In urban areas, the high human population density favors the mosquito-human contact and, therefore, the chance of becoming infected, especially when finding a large portion of the susceptible population^33^. In addition, in urban and rural locations, the presence of breeding sites in environments for human interaction, precarious services, inadequate infrastructure, the increase in the production of nonorganic waste, and the greater migratory dynamics explain the greater risk of the occurrence of arboviruses^34^.

The positive association found between the coefficient of arboviruses detection and irregular waste disposal sites is consistent with other findings and may be justified by the possibility of waste becoming potential breeding grounds for *A. aegypti*. A survey conducted by the IBGE on basic sanitation shows that Dengue was the most reported disease by municipalities and that it was associated, among other factors, with garbage accumulated in homes and on the streets^35^.

A study evaluated the association and the impact of waste collection on cases of Dengue fever in Recife, state of Pernambuco, Brazil, from January 2013 to February 2015 and identified a strong negative correlation between the monthly uncategorized weighing of garbage and the collection of household waste and the total number of confirmed cases of the disease^4^. In that same study, it was found that the collection of construction debris and waste, selective collection and tires were also negatively associated with cases of Dengue fever in the municipality, showing that regular waste collection and the reduction of household waste, with actions to optimize routes and increase collection frequencies by the public authorities, would reduce cases of the disease. The lower coverage of selective collection was also associated with the highest number of registered cases of Dengue in the period from 2007 to 2016, in municipalities of the state of Minas Gerais, Brazil^36^.

The irregular disposal of waste in several locations increases the problem, especially when these places are located in the suburbs. Some studies have pointed out this fact as being largely responsible for the high number of diseases such as Dengue^4,13^. Although these conditions are not directly linked to the occurrence of arboviruses, they can provide favorable conditions for the larval development of *A. aegypti* ^12^. As urban expansion takes place unrelated to social policies and housing infrastructure, a complex health framework is created, in which limited access to basic sanitation services and inadequate housing have negative impacts on the population’s morbidity and mortality profiles^37^.

In this study, we identified no significant relationship between the coefficient of arboviruses detection and SDI. It is possible that this variable used in the spatial regression model did not capture the entire spatial pattern, probably because a global regression model was used, which considers the spatial process underlying the data analyzed in a single parameter, that is, stationary. However, we use census data, and these can produce several spatial patterns that are not identified in a single parameter. We suggest new investigations, involving models that consider local spatial effects, considering that the parameters vary in space^22^.

Despite the relevant results of our study, there were some limitations. The use of secondary data from the 2010 Census with lack of data on variables of interest (education and income information) is justified by the lack of timely availability of data from the 2020 Census for the research. We were unable to include variables related to disease transmission such as vector spatial density, breeding productivity and building infestation index^38-40^. The difficulty in accessing vector data limits the interpretation of the findings in relation to the spatiotemporal distribution of the coefficients of arboviruses incidence and their correlation with solid waste disposals.

The study advances knowledge and aligns with other research on the positive correlation between the frequency of urban arboviruses and the increase in solid waste disposal areas. The spatial analysis by census tract is noteworthy, being, therefore, more accurate regarding the risk of getting sick according to the territories, and based on a large number of analyzed cases.

Finally, the control of arboviruses is a major challenge for managers, as it goes beyond the limits of health management and requires integrated action with other sectors and services such as urban cleaning, infrastructure, and solid waste management.
